# The Relationship Between 5-Hydroxytryptamine and Its Metabolite Changes With Post-stroke Depression

**DOI:** 10.3389/fpsyt.2022.871754

**Published:** 2022-04-26

**Authors:** Simeng Gu, Zhengming He, Qiuyue Xu, Jie Dong, Tingwei Xiao, Fei Liang, Xianjun Ma, Fushun Wang, Jason H. Huang

**Affiliations:** ^1^Department of Psychology, Jiangsu University Medical School, Zhenjiang, China; ^2^Institute of Brain and Psychological Science, Sichuan Normal University, Chengdu, China; ^3^Department of Nurse, Nanjing University of Chinese Medicine, Nanjing, China; ^4^Section of Brain Diseases, Department of Neurology, Lianyungang Hospital of Chinese Medicine, Affiliated Hospital of Nanjing University of Chinese Medicine, Lianyungang, China; ^5^Department of Neurosurgery, Baylor Scott & White Health, Temple, TX, United States; ^6^Department of Surgery, Texas A&M University College of Medicine, Temple, TX, United States

**Keywords:** post-stroke depression, 5-Hydroxytryptamine, monoamine hypothesis, three primary emotions, emotional intervention

## Abstract

Post-stroke depression (PSD) is the most common and serious sequelae of stroke. Approximately 33% of stroke survivors were affected by PSD. However, many issues (e.g., incidence, diagnostic marker, and risk factor) related to PSD remained unclear. The “monoamine hypothesis” is a significant hypothesis for depression, which suggests that three monoamines play a key role in depression. Therefore, most current antidepressants are developed to modulate the monoamines on PSD treatment, and these antidepressants have good effects on patients with PSD. However, the potential mechanisms of three monoamines in PSD are still unclear. Previously, we proposed “three primary emotions,” which suggested a new model of basic emotions based on the three monoamines. It may provide a new way for PSD treatment. In addition, recent studies have found that monoamine-related emotional intervention also showed potential effects in the treatment and prevention of PSD. This study discusses these issues and attempts to provide a prospect for future research on PSD.

## Introduction

Post-stroke depression (PSD) is a common and serious complication after stroke, which is often regarded as the inevitable reaction toward stroke-related disability (1). A recent meta-analysis reported that the incidence of PSD within the first 5 years after stroke ranged from 25 to 33% (2). PSD adversely affects recovery and the life quality in patients with stroke. Evidence suggests that PSD is related to a large number of adverse health outcomes, such as increased morbidity, disability, and mortality (3–5). However, as the mechanisms of PSD diagnosis are unclear, the specific critical periods for most interventions are still uncertain and most antidepressants used for PSD have been reported to have serious side effects, until present some of the patients remain untreated or not be adequately treated (6).

At present, the main therapeutic approach to PSD is essentially pharmacological (7), and the most commonly used pharmacotherapeutic agents for treating PSD are antidepressants (1). Three monoamines, namely, dopamine (DA), 5-hydroxytryptamine (5-HT), and norepinephrine (NE), play key roles in the etiology and treatment for major depressive disorders (MDD) (8). The monoamine hypothesis assumes that depression is associated with low levels of monoamines, especially DA, 5-HT, and/or NE (8, 9). So the major antidepressants for MDD are designed to increase monoamine transmission either by inhibiting neuronal reuptake or by inhibiting degradation (8, 10).

In addition, these three monoamines might be the primary substrate for emotions (11). Previously, we have proposed the “three primary color model” of basic emotions based on the three monoamines Wang et al. (2020). In the hypothesis, we suggested that all emotions are composed of some basic emotions, such as happiness, sadness, and anger and fear, which are subsided, respectively, by the three neurotransmitters: DA (happiness), 5-HT (disgust), and NE (fear and anger) (12). Depression and other affective disorders (such as PSD and anxiety) are related to the dysfunctions of the monoamine system (13, 14).

It might be easy to suggest that the etiology of PSD may be the ischemic lesions caused by stroke interrupting the projections ascending from the midbrain and brainstem, leading to a decreased bioavailability of the biogenic amines, including DA, 5-HT, and NE (4, 7). Even though traditional antidepressants are the first-line treatment used for PSD, the mechanisms of PSD are still unclear (4). Therefore, this study aims to review the relationship of PSD with three monoamines and emotions. First, we briefly introduced the incidence, risk factors, and diagnosis of PSD. Then, we reviewed the application of three monoamines in the treatment of PSD drugs and the “three primary color model” of basic emotions. Finally, we summarized the advantages of psychological therapy in recent years and posted some suggestions for the pharmacology and psychotherapy of PSD.

## Post-Stroke Depression

Stroke and depression are two leading causes of disability worldwide (6, 15). They not only negatively affect patients' life quality but also lead to socioeconomic burden (15, 16). PSD is the most frequent and important neuropsychiatric consequence of stroke (17). According to a report by World Health Organization (WHO), approximately one-third of the 15 million patients with stroke (2) suffer from PSD every year globally (18). Despite the similarities between PSD and MMD, there are some significant differences between them (4, 15). First, PSD is a complication of stroke, which is closely linked to vascular injury (19), while MMD is majorly due to monoaminergic systems. Second, PSD and MMD are different in symptoms in that PSD tends to have more severe cognitive impairment than MMD but less anhedonia and disturbances in sleep and cyclic functions than MMD (20, 21). Third, patients with PSD have a higher prevalence of physical disability, which may be related to stroke (22). Therefore, the clinical characteristics of PSD are not identical to those of MMD, and PSD needs to be specifically discussed.

## Incidence Of PSD

As a common stroke complication, PSD has been investigated by many scientists in many countries around the world (23). In addition, many meta-analysis studies have investigated the incidence and etiology of PSD (2, 24, 25). In his pioneering studies of PSD, Hackett et al. (26) conducted a systemic review and meta-analysis, which included 17,934 patients from 20 studies and revealed a pooled frequency estimation of PSD of 33%. Hackett et al. (2) updated the systematic review with a meta-analysis about the frequency of PSD in the next 10 years. They revealed that the pooled frequency was estimated to be 31%, which was consistent with the results found in a 10-year earlier review. Recently, a new study reported the incidence of PSD within the first 5 years following stroke to be 39–52%, which is far higher than the incidence of MDD (about 4.4% of the world's population) (27).

Similar to Hackett et al., Ayerbe and his colleagues revealed a similar pooled frequency of PSD of 29% and a cumulative incidence of 39–52% within 5 years of the stroke (24). The interesting finding of this research is that the frequency of PSD remained quite consistent for the first year but then started to decline. However, another study has provided an opposite result as to the time course of PSD. Werheid et al. (28) reported a two-phase pathogenetic model of PSD based on 10 prospective longitudinal studies, which revealed a rise in the incidence of PSD within the first 6 months, a slight drop at about 1 year, and a new increase within the second year following a stroke.

In a recent study, Eman et al. (29) used DSM-IV TR as diagnosis criteria of depressive disorders, they found that the frequency of PSD was 36.9%, and 21.4% of which had MDDs, meanwhile 15.5% had minor depressive disorders. Even though these studies have provided the frequency and severity of PSD, still there exist one limitation in these studies because there were no standard diagnostic criteria for specific mood disorders in most studies (16). In other words, these meta-analyses did not distinguish major depression from other forms of depressive disorders occurring after stroke (23). In addition, an obvious finding was that there were differences in the results of different PSD incidence studies due to the differences in sample size, geographical location, the selection of patients, etc. (30).

## Diagnosis of PSD

A longstanding problem was that a vast majority of patients with stroke are not screened for PSD (15) because PSD was confused with many mood disorders in symptoms, such as post-stroke apathy (PSA) (31) and catastrophic reaction (32). PSA is generally defined as a disorder of diminished motivation caused by a stroke (31). The symptoms of PSA are loss of interest, diminished emotional response, and loss of initiative (33), which are quite similar to those of PSD. In addition, based on physiology, both PSD and PSA are related to fronto-striatal circuit dysfunction and small vessel ischemia (34, 35). A catastrophic reaction is also a common emotional reaction after stroke. The definition of catastrophic reaction is an intense emotional reaction to the inability to perform tasks after neurological damage (36), which is characterized by severe frustration, sadness, anger, or aggression (15). Although these symptoms are similar to those of post-stroke diseases, the treatments are quite different. For example, substantial evidence shows that PSA is better treated by psychotherapy interventions instead of antidepressants (37), but antidepressants in fact have shown good therapeutic effects in PSD treatment (7). Therefore, the diagnosis and treatment of PSD are particularly important. The diagnosis and screening of PSD mainly use the traditional depression scales (38), such as Hamilton Depression Rating Scale (HAM-D), Beck Depression Inventory (BDI), and Hospital Anxiety and Depression Scale (HADS) (39). We summarized the main PSD diagnosis instruments in Table 1 according to recent reports; however, Nick et al. (40) conducted a meta-analysis on these diagnostic methods for PSD. They found that all the tools used in the clinics were not so correct for case findings. In all these scales, the Center of Epidemiological Studies-Depression Scale (CESD), HAM-D, and the Patient Health Questionnaire (PHQ-9) showed the best results. PHQ-9 is the shortest of these options, with only nine questions based on the DSM-IV criteria for MDD (41). As a result, the PHQ-9 is one of the fastest and most practical tools that can be administered in the screening and diagnosis of PSD (15) (Table 1).

**Table 1:** Main tools to screen and diagnose the PSD.

**Scales**	**Full name**	**Authors**	**Diagnostic criteria**	**Sensitivity (95% CI)**	**Specificity (95% CI)**
DSM-IV	Diagnostic and statistical manual of mental disorders	American Psychiatric Association	∙presence of depressed mood or anhedonia ∙symptoms are pathophysiologically related to the stroke ∙symptoms are not better explained by other psychiatric disorders ∙disturbance does not occur exclusively in the presence of delirium ∙symptoms cause significant distress or impairment		
PHQ-9	9-item Patient Health Questionnaire	Spitzer RL	Self-rating scale; all items are graded from 0 to 3; score greater than 4 is diagnosed as having depressive symptoms	0.86 (0.70 to 0.94)	0.79 (0.60 to 0.90)
HAMD	Hamilton Depression Rating Scale	Hamilton	Two trained assessors conduct joint inspections on the assesses; score greater than 7 is diagnosed as having depressive symptoms	0.82 (0.69 to 0.90)	0.75 (0.62 to 0.84)
CES-D	Center of Epidemiological Studies-Depression Scale	Sirodff	20 items; self-rating scale; according to the frequency of the corresponding condition or feeling in the past 1 week; it focuses more on the emotional experience of the individual; score greater than 15 is diagnosed as having depressive symptoms	0.64 (0.48 to 0.78)	0.85 (0.52 to 0.97)
BDI	Beck Depression Inventory	Beck AT	13 items; all items are graded from 0 to 3; score greater than 4 is diagnosed as having depressive symptoms	0.90 (0.62 to 0.98)	0.55 (0.19 to 0.86)
HADS	Hospital Anxiety and Depression Scale	Zigmond AS and RP Snaith	Divide into anxiety subscale and depression subscale with 7 items each; score greater than 10 is diagnosed as having depressive symptoms	0.87 (0.46 to 0.98)	0.73 (0.65 to 0.79)
MADRS	Montgomery-Asberg Depression Rating Scale	Montgomery SA, Asberg M	10 items; all items are graded from 0 to 6; score greater than 12 is diagnosed as having depressive symptoms	0.85 (0.78 to 0.90)	0.79 (0.70 to 0.86)
GDS	Geriatric Depression Scale	Brank	30 items; self-rating scale; suitable for the elderly; score >20 is diagnosed as having depressive symptoms	0.81 (0.65 to 0.91)	0.77 (0.62 to 0.82)

## Risk Factors Of PSD

Depression is a common symptom following a stroke; however, the risk factors and predictors are yet to be delineated (1). The benefit of understanding PSD risk factors is beneficial to the prevention and treatment of this disease. Many studies during the past decades have reported many causing factors for PSD. The main factors are summarized in the following sections.

### Stroke-Related Factors

A series of studies have found that the type, severity, and lesion location of stroke were related to the PSD (30). Jørgensen et al. (42) conducted a large sample study by collecting data from 157,243 patients with stroke between January 2001 and December 2011. They reported that patients with ischemic stroke had a higher incidence of PSD than patients with hemorrhagic stroke. In another study, Vataja et al. (43) also found that patients with PSD had more sites and a larger volume of infarcts. However, another study did not find different rates of PSD based on the type of stroke (44).

A lot of studies have provided evidence for the relationship between stroke severity and PSD incidence (24, 45, 46). One meta-analysis study of PSD by Hackett et al. (26) found that there was a positive correlation between stroke severity and PSD. Recently, a multiple regression analysis from Taiwan also found a correlation between the severity of stroke and the incidence of PSD (46). Another study by Jørgensen et al. (42) also found that a higher depression score was significantly associated with PSD, regardless of gender.

In addition, the lesion location of the brain was strongly associated with PSD. In a series of studies (23, 47, 48), Robinson and his colleagues revealed that patients with stroke in the left hemisphere had a higher incidence of PSD and the severity correlated significantly with the proximity of the lesion to the frontal pole. Meanwhile, Starkstein et al. (49) found that the location of subcortical lesions had a greater influence than cortical lesions on PSD. Similarly, in patients with subcortical damage, the closer the lesion to the frontal lobe, the more severe the PSD. Therefore, the frontal pole may play a key role in the severity of PSD. In other ways, Jorge et al. (50) found that focal brain stimulation using repetitive transcranial magnetic stimulation is only effective when it is applied to the left dorsolateral prefrontal cortex in patients with vascular depression. Robinson (23) also considered that PSD is associated with left frontal or left basal ganglia lesions within 2 months of a first clinical stroke. Therefore, left frontal or left basal ganglia lesions may be used as the screen basis of PSD.

### Demographic Factors

Similar to MDD, many demographic factors, such as sex, age, and history of psychiatric illness, are related to the PSD. During the past decades, there was no agreement on sex as a risk factor for PSD. Some studies identify female sex as a risk factor for PSD. In a meta-analysis study of the risk factors for PSD, Shi et al. (17) found that sex (female) was significantly associated with PSD [OR = 1.77, 95% CI = 1.26–2.49]. This result was also reported in other studies (46, 51, 52). However, a systematic review by Ryck (44) found that gender was not a significant risk factor for PSD in 13 out of all 21 studies.

Age was another factor that yielded the most controversial results. In a study of 216 patients with ischemic stroke, Li et al. (53) revealed a difference in age between patients with PSD and patients without PSD. Carota et al. (54) also found the association between PSD and age. However, Ryck et al. (44) revealed that age was not associated with PSD in 16 studies. Therefore, the relationship between age and PSD is still unclear.

Finally, the history of psychiatric disorders was also associated with PSD, particularly MDD and anxiety disorders. A meta-analysis study by Ried and his colleagues found the rate of PSD was found to be 5–6 times higher among those with pre-stroke depression (55). A recent study has also revealed depression before stroke notably increased odds of PSD (56). Anxiety disorder is also a risk factor for PSD. De Ryck reported that a personal history of anxiety was a significant risk factor in some studies (44).

### Social Support

Apart from the above risk factors, social support is also associated with the PSD. But, the available studies concerning PSD and social support are contradictory (23). A systematic review of the relationship between social support and PSD reported that some factors (such as family life, friends, acquaintances, and social participation) of social support were associated with PSD, and lack of social support may cause more severe PSD symptoms (57). Other studies have reached similar results (18, 58). Even though a lot of evidence indicated that social support was related to PSD, Jessica et al. (59) reported that living conditions and marital status have not been consistently associated with PSD.

## Monoamine Transmitters In The Treatment Of PSD

In their study performed 2,500 years ago, Hippocrates and Galen suggested that individual differences are due to fluid components in the body, and that a balanced mixture of these vital chemicals can induce at least four kinds of temperaments: choleric (aggressive), melancholic (depressive), phlegmatic (fear and social detached), and sanguine (cheerful) (60). The further that research in the neurochemistry of emotionality advances, the more that neurochemical systems are linked to emotional regulation (61). In fact, dysregulation in practically all neurochemical families, especially monoamines, hormones, neuropeptides, opioid receptors, and transcription factors, appears to contribute to PSD (62). There are two main theoretical views about the determinants of PSD. One of them focuses on brain locations such as the amygdala and hippocampus, prefrontal cortex, and hypothalamus. Another one emphasizes neurochemicals such as disruption of biogenic amine neurotransmission and release of proinflammatory cytokines (63). The monoamine hypothesis assumed that PSD was related to abrupt damage of cortical circuits involved in mood regulation and monoamine production (64). Dopamine (DA), 5-HT (5-HT) and norepinephrine (NE) are the three main monoamine transmitters in emotion regulation (11), which play a key role in antidepressant drugs for PSD treatment. In the following section, the roles of 5-HT, DA, and NE in the treatment of PSD are discussed.

### Monoamine Hypothesis

The “monoamine hypothesis” of depression originated from early clinical observations (8), which posited that depression was caused by an alteration in one or more of the monoamines (65). Robinson (48) assumed that ischemic lesions may interrupt the biogenic amine-containing axons ascending from the brainstem to the cerebral cortex and lead to a decreased availability of monoamines (5-HT, DA, and NE) in limbic structures of frontal, temporal lobes, and basal ganglia. Monoaminergic neurons in the midbrain dynamically alter their firing patterns, which were associated with motivation-related behavior in animal studies (66). Motivation-related behavior included salience, reward and punishment learning, incentive processing, decision-making, goal-directed behavior, and anxiety (67). Recent studies have revealed three different monoaminergic dynamics that regulate diverse aspects of motivation-related behavior (68–71). Therefore, it appeared that specific aspects of motivation-related behavior were regulated by distinct synaptic and cellular mechanisms in specific brain regions that underlie the transient and sustained effects of the monoamine signaling (66). The different neural systems of three monoamines may involve different symptoms of depression (mood, cognition, and pain). Serotonergic (5-HT) neurons originate from the median raphe nucleus and innervate the limbic system, prefrontal cortex, and other related structures involved in the regulation of mood (4). In addition, 5-HT projected to the basal ganglia has been confirmed to be associated with motor control (72). Dopaminergic projections originated from the ventral tegmental area (VTA) and substantia nigra (SN), reaching different regions of nucleus accumbens (Nac), had been proven to be related to reward and aversion (73). Norepinephrinergic neurons originated from the locus coeruleus (LC) project to the limbic system to participate in the regulation of emotional arousal (74). Furthermore, the monoaminergic descending pathways projecting through the dorsolateral spinal column played an important role in the regulation of pain.

Since the 1950s, reserpine has been found to inhibit vesicular monoamine transporters and deplete brain monoamines, which provided evidence for the role of monoamines in the treatment of depression. In 1959, the Food and Drug Administration (FDA) approved imipramine for the treatment of MDD, which established the class of drugs called tricyclic antidepressants (TCA) as the first class of drugs to target monoamines. Later, selective 5-HT reuptake inhibitors (SSRIs) and 5-HT and norepinephrine reuptake inhibitors (SNRIs), which are based on the “monoamine hypothesis,” were approved for depression in 1987 and 1993, respectively. In recent years, some drugs targeting the glutamate system (such as ketamine) showed good effects (75). In all, the introduction of TCAs and monoamine oxidase inhibitors based on the monoamine hypothesis revolutionized the treatment of depression. Since then, most of antidepressants have been developed by primarily acting through modulation of monoaminergic neurotransmission (76). Even though the monoamine hypothesis alone was no more generally accepted (16), the current main treatment of PSD drugs is still based on the monoamine hypothesis (77).

### Serotonin (5-HT)

5-Hydroxytryptamine is a significant neuromodulator with unique neuroplastic capabilities (78). The main gathering area for 5-HT neurons is the dorsal raphe nucleus (DRN). The 5-Htergic neurons of the DRN send projection to the entire brain and throughout the neuraxis and receive major inputs from the hypothalamus, amygdala, midbrain, and anterior neocortex (66). There are 14 types of serotonergic receptors, which can be divided into seven main families according to differently coupled G-proteins (79). Each group of receptors may have different functions. For example, 5-HT_1A_ and 5-HT_1B_ receptors are associated with anxiety (80) and reward behaviors (81); 5-HT_2A_ receptors are correlated to appetite control, thermoregulation, and sustained attention (82); 5-HT_3_ receptors are related to aggression behaviors (83); and 5-HT_4_ receptors affect memory, depression, and feeding (84).

**Figure 1:**
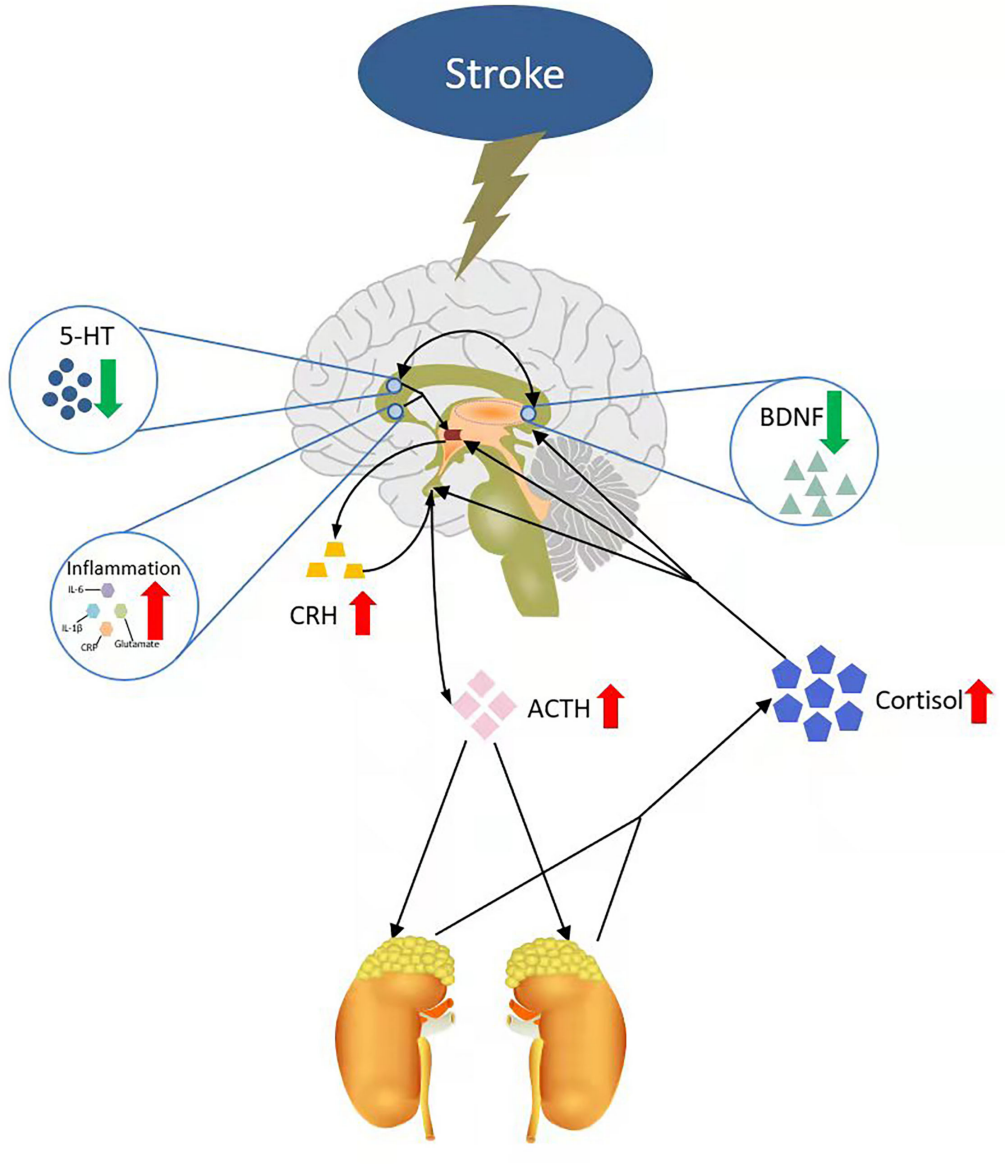
A neural mechanism model of PSD. IL-6, interleukin-6; IL-1β, interleukin-1β; CRP, C-reactive protein; CRH, corticotropin-releasing hormone; ACTH, adrenocorticotropic hormone; 5-HT, 5-hydroxytryptamine; BDNF, brain-derived neurotrophic factor.

In addition, abundant evidence has justified the role of 5-HT in depression (85), as well as in patients with PSD (86). Furthermore, 5-HT levels can be affected by three neurobiologically related factors of PSD: increased inflammation and trauma, decreased cerebral brain-derived neurotrophic factor (BDNF), and dysregulation of the hypothalamus-pituitary-adrenal (HPA) axis. Raison et al. (87) reported that the metabolisms of 5-HT are affected by the central nervous system (CNS) inflammatory response (88). A peripherally administered cytokine could activate a CNS inflammatory response in humans that interacted with 5-HT metabolism, which was associated with depression. The association of BDNF and 5-HT also showed a special feature in depression (89). BDNF injected into the midbrain increased the level of 5-HT and also enhanced the expression of genes encoding 5-HT_1A_ and 5-HT_2A_ receptors. These changes can only be observed in depression mice but not in nondepression mice (90). In patients with PSD, 5-HT and other monoamine (DA and NE) release might be affected by abnormal HPA axis activity after stroke. Therefore, Guo et al. (30) posted a model of the PSD mechanism based on four main hypotheses of PSD: the monoamine hypothesis, HPA hypothesis, neurotrophic hypothesis, and inflammation hypothesis. The model considered that stroke could trigger a robust inflammatory response and severe monoamine system damage in the injured brain region (91). Then, this change would increase the activity of HPA, which works through the following processes. First, the inflammatory response and the 5-HT decrease made the paraventricular nucleus of the hypothalamus release more corticotropin-releasing hormone (CRH), which stimulated the pituitary to release more adrenocorticotropic hormone (ACTH). The increase in ACTH release causes an increase in glucocorticoid synthesis and release in the adrenal cortex (92). Glucocorticoid is also called cortisol in the human body, which is the major component of the HPA axis. Many studies have shown that the cortisol levels were higher in depression patients (93, 94). An increase in cortisol, in turn, could lead to a decrease in BDNF, which played a key role in the emotion system. On the one hand, lacking cerebral BDNF contributed to the development of negative mood states (95). On the other hand, BDNF was closely associated with 5-HT, and the functional activity of the 5-HT system was linked with depression and suicide (89). 5-HT decrease in the limbic system and cerebral cortex might be an important factor for depression in patients with stroke. Therefore, 5-HT and its receptors can be used as a biomarker for PSD (Figure 1).

Furthermore, the major antidepressant drugs mainly target 5-HT and its receptors. Currently, there are three main types of antidepressant drugs, including tricyclic antidepressants (TCAs), selective 5-HT reuptake inhibitors (SSRIs), and 5-HT and norepinephrine reuptake inhibitors (SNRIs) (96). The first-line therapeutic treatment for PSD is using antidepressants, as can be seen in a recent meta-analysis, which confirmed that antidepressant drugs had a significant effect in the treatment of PSD vs. placebo (97). TCAs are a group of traditional antidepressant drugs, and some drugs of this kind are still used in PSD treatment, such as amitriptyline and nortriptyline, which are 5-HT_2_ receptor antagonists. Xu et al. (97) found a significant advantage of TCAs over placebo in a meta-analysis study. However, both amitriptyline and nortriptyline have been reported to have serious side effects. Some elderly patients with stroke showed orthostatic hypotension, cardiac arrhythmia, glaucoma, or prostate hyperplasia after using the two drugs (98). SSRIs and SNRIs are two groups of new antidepressant drugs, which is introduced after the 1980s (7). Nowadays, there are at least 6 SSRI drugs (fluoxetine, paroxetine, fluvoxamine, sertraline, citalopram, and escitalopram) and 3 SNRI drugs (i.e., milnacipran, duloxetine, and duloxetine) available in PSD treatment. Anyway, a series of studies have shown that the SSRI drugs also had great effects on PSD treatment (99–105), such as gastrointestinal symptoms, headache, sexual dysfunction, and insomnia (7).

In addition, using SSRIs may increase mortality in patients with stroke (24). However, these studies did not reach a consensus result, even opposite results (106, 107). Compared with SSRIs, SNRIs may be useful in improving painful physical symptoms due to their noradrenergic action. Some studies have also found that SNRIs show a great effect on PSD prevention and treatment (103, 108, 109). In addition to these three types of drugs, some new antidepressant drugs also target 5-HT receptors and show great effects on patients with PSD. For example, vortioxetine, a new antidepressant with multimodal activity, shows great therapeutic effects on cognition. It can act on multiple 5-HT receptors, including 5-HT_1A_, 5-HT_1B_, 5-HT_1D_, 5-HT_3_, 5-HT_7_, and 5-HT transporter (SERT) (110). In addition, vortioxetine shows fewer side effects than current first-line antidepressants. In all, these studies showed that antidepressant drugs targeting 5-HT can play a role in the treatment of PSD (Table 2).

**Table 2:** Main 5-HT drugs and their receptors for PSD.

**Drugs**	**5-HT receptors**	**Clinical application**	**References**
Fluoxetine	5-HT2C (-)	depression, premenstrual dysphoric disorder, hypochondriasis, bulimia nervosa	(111)
Paroxetine	5-HT2C (-), 5-HT2A (-)	depression, PTSD, OCD, generalized anxiety disorder, premenstrual dysphoric disorder	(112, 113)
Fluvoxamine	5-HT1A (-)	anxiety disorders, schizophrenia, delusional depression	(114, 115)
Sertraline	5-HT2C (-)	major depression, panic disorder, OCD, PTSD	(116)
Citalopram	5-HT3 (-), 5-HT1A (-), 5-HT2C (-)	major depression, OCD	(117, 118)
Escitalopram	5-HT1A (+)	depression, anxiety disorder	(119)
Amitriptyline	5-HT2 (-)	schizophrenia,	(120)
Nortriptyline	5-HT2 (-)	depression	(121)
Clomipramine	5-HT1A (+), 5-HT1B (-)	OCD, major depression	(122)
Milnacipran	5-HT1A (-)	major depression	(123)
Duloxetine	5-HT (-)	generalized anxiety disorder, major depression	(124)
Mirtazapine	5-HT2A (-)	depression, PTSD	(125, 126)
Venlafaxine	5-HT1B (-)	major depression, OCD	(127)
Doxepin	5-HT2A (-), 5-HT2C (+)	insomnia	(128)

### Dopamine and NE

Dopamine and NE are two other monoamines and also play a key role in the emotion system. The dopaminergic system is a unique modulatory system in the brain as it has discrete projections to specific brain regions, including motor behavior, cognition, and emotion (110). Unlike 5-HT or NE, separate groups of DA neurons project to different brain regions. Different groups of DA neurons project to different brain regions to moderate and regulate different behaviors and functions (129). Dopaminergic neurons are mainly located in the VTA and SN (130). Dopaminergic neurons in these two areas project to the reward-related Nac and ventral striatum (VS), which is called the mesolimbic DA system (22). In addition, the dopaminergic neurons in the lateral SN primarily project to the dorsomedial striatum and participate in the formation of motor learning and habit behavior (131). The functions of DA are mainly mediated by DA receptors, which are composed of five different but closely related G protein-coupled receptors, D1-like (D1 and D5) and D2-like (D2, D3, and D4) receptors (Beaulieu, Gainetdinov, & Sibley, 2011). D1-like receptors can enhance the activity by activating the Gαs/olf family, but the D2-like receptors activate Gs/ol family and inhibit the activity (79, 132). More and more studies have shown that dopaminergic system dysfunction is linked to the pathology of depression (133–136). Anhedonia and amotivation are two main symptoms seen in depression, which are related to dysfunctions in the dopaminergic system (137). Animal models of depression showed stress-induced impairments of VTA dopaminergic neurons are related to the increasing susceptibility of depression in rats (138), which is due to stress increased activity of dopaminergic neurons in the circuit of the hippocampus—VS-ventral pallidum. However, increased activity in the ilPFC-amygdala-ventral pallidum circuit caused a compensatory, long-duration downregulation of the VTA. The downregulation of the VTA was maintained after stress, which might be the reason for anhedonia and depression (139). Therefore, we could speculate that stroke led to serious monoaminergic system damage, which led to reduced release of VTA dopamine to the reward-related Nac and VTA, and thus anhedonia and depression. In addition, antidepressant drugs targeting DA and its receptors also showed great benefits in PSD treatment. For example, fluoxetine and paroxetine, two of the most commonly used drugs in PSD treatment, could prevent the degeneration of nigrostriatal dopaminergic neurons (140). A recent study has revealed that SNRIs achieve a fast antidepressant effect by elevating the DA concentrations in the mPFC and the Nac (96). Furthermore, a new antidepressant drug, bupropion, which primarily acts through the NE transporter and DA transporter, shows a significant therapeutic effect (141).

Norepinephrine, a catecholamine neuromodulator, projects to all the brain regions except some dopaminergic neuron regions, such as the striatum, globus pallidus, NAc, and SN (142). NE is mainly released from neurons originating from locus coeruleus (LC), a small nucleus situated in the pons of the brainstem. The LC-NE system has long been considered to be critical in arousal (71). NE exerts its effects through binding to G-protein coupled α-adrenergic receptor (α-AR) and β-adrenergic receptor (β-AR). A-AR receptors can be divided into two families: α1 and α2. Each of them has three subtypes: α_1A_, α_1B_, and α_1D_; α_2A_, α_2B_, and α_2C;_ while β-Ars has two groups: β1and β2 (143). NE has the highest affinity for the α2 receptors and the lowest affinity for β adrenergic receptors. In addition, α1 receptor stimulation has been found to enhance excitatory processes in many brain regions (144). Animal models offered the evidence that reduction of the levels of presynaptic NE, such as 5-HT, or DA, plays a key role in the pathophysiology of depression (145). In all, the LC-NE system is also related to the low arousal state of depression (74). A meta-study showed a significant correlation between baseline 3-methoxy-4-hydroxyphenylglycol (sMHPG) levels and Beck Depression Inventory (BDI) score, and sMHPG was the major NE metabolite in the cerebrospinal fluid (146). Leonard et al. (147) proposed a model about the relationship between NE and depression. They proposed that chronic stress activated the release of corticotropin-releasing factor (CRF), leading to the increased release of pro-inflammatory cytokines, prostaglandins of the E series, and nitric oxide, which influenced the central neurotransmitter function. If these changes persisted, they may contribute to the degenerative changes in noradrenergic neurons, which would lead to depression. In patients with stroke, stroke might change NE levels and thus PSD. In terms of depression medications, SNRIs showed faster antidepressant effects than SSRIs, and the underlying mechanisms of faster antidepressant effects of SNRIs may be related to NE (96). In all, SNRIs showed a great effect in improving painful physical symptoms due to their noradrenergic action (7). A meta-study showed in recent clinical studies that NE may play an important role in aberrant regulation of cognition, arousal, and valence systems that are associated with depression (143).

### Monoamine and Related Chemicals

**Figure 2:**
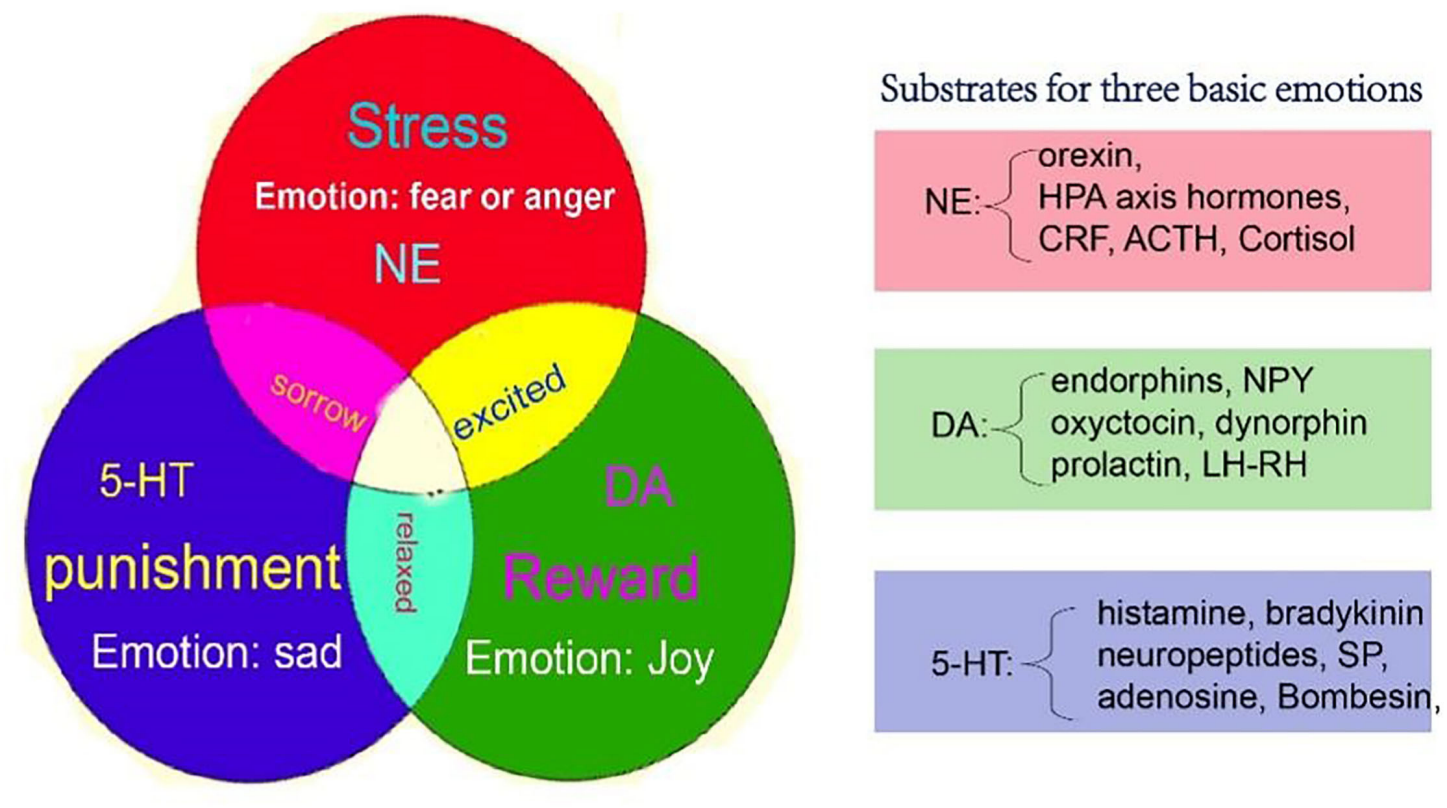
A new model for basic emotions, which might be the neural mechanisms of PSD. The monoamine (NE, DA, and 5-HT) might be the substrate for basic emotions, which include joy, sadness (disgust), and fear (anger). [A figure from our previous paper, (61)]. NE, norepinephrine; DA, dopamine; 5-HT, 5-hydroxytryptamine.

Even though the monoaminergic systems are implicated in the regulation of basic emotions, there is a functional overlap of neurochemical systems related to PSD. The neurochemicals involved in PSD can be divided into two groups: neuromodulators and neuropeptides (Table 3). The neuromodulators are small molecules, such as monoamines, and have specific functions, such as joy, disgust, and fear, like the three primary colors. While the peptides such as oxytocin, orexin, and neuropeptides are more complex and carry more flexible functions, which might be secondary to the neuromodulators. Anyway, the monoamines and other secondary neurochemicals can interact with each other to produce different kinds of emotions (Figure 2). The difference might be that the neuropeptides are involved in more specific functions, such as thirst, hunger, and pain (Table 3).

**Table 3:** Monoamine and chemicals for PSD.

**Neuropeptide**	**Emotional feelings**	**References**
Substance P	Pain and anger	(148, 149)
Angiotensin	Thirst	(150)
Oxytocin	Orgasm, maternal feelings	(151, 152)
ACTH	Stress	(153)
Insulin	Energy	(154)
Vasopressin	Male sexual arousal, dominance	(155, 156)
Bradykinin	Pain	(157)
CCK	Satiety, disgust	(158, 159)
Prolactin	Maternal and love	(160, 161)
TRH	Playfulness	(162)
LH-RH	Female sexual arousal	(163)
Bombesin	Satiety-disgust	(164)
Neurotensin	Seeking	(165)
Enkephalin	Pain	(166, 167)
Endorphin	pleasure	(168, 169)
DSIP	Boring-disgust	(170)
Dynorphin	Hunger	(171)
CRF	Panic, anxiety	(172, 173)
NPY	Hunger	(174)

## The Theory of Three Primary Emotions

Although the “monoamine hypothesis” proposed that the mood symptoms of depression were mainly related to decreased levels of monoamines, the relationship between monoamine transmitters and emotion was never clarified. Nowadays, there are two widely accepted theories in emotional studies: basic emotion theory and dimensional theory (175). The basic emotion theory suggests that all emotions are composed of a limited number of emotions (11). Basic emotions have evolutionarily preserved biological and social functions (175). After many experimental studies, Ekman (176) suggested that people have six basic emotions: joy, sadness, fear, anger, disgust, and surprise. Robert Plutchik proposed eight primary emotions in a color wheel: anger, fear, sadness, disgust, surprise, anticipation, trust, and joy (175). In recent years, Jack et al. (177) proposed four basic emotions: fear, anger, joy, and sadness. The dimensional theory proposes that emotions could be defined by some different dimensions, and all emotions could be defined as a combination of these dimensions (178). The dimensional theory was first proposed by Wundt, who suggested that emotion had three independent dimensions: pleasant-unpleasant, tension-relaxation, and excitation-calm (179). The most famous dimensional theory was proposed by Russell et al., who invented the circumplex, which is composed of two dimensions: hedonic (pleasure-displeasure) and arousal (rest-activated). They proposed that all emotions could be arranged in a circle, and the different locations of each emotion in the circle reflected varying amounts of hedonic and arousal properties (180). Even though both theories were supported by more studies, no previous reports have connected emotion with neurotransmitters. Wang et al. (61, 79, 178, 179) posted a new theory of three primary emotions, which not only compromised both basic emotion theory and dimensional theory but also associated with neurotransmitters, especially monoamines, with emotions.

Basic emotions are instinctive, primitive, and developed throughout evolution (175), and each basic emotion should have a specific neural basis. Therefore, Wang et al. (12, 61, 170) proposed the theory of three primary emotions *via* a large number of basic emotional studies. They proposed three basic emotions: joy, disgust, and fear (anger), which were subsided, respectively, by the three monoamine neurotransmitters: DA, happiness; 5-HT, sadness; and NE, fear (anger). Fear and anger are twin emotions that are like two sides of the same coin (61). Fear and anger are associated with unanticipated ways things happen: fear is associated with uncertainty about the situation; and anger is related to trying to control the situation (181), which can induce the individuals to generate the so-called fight or flight response (182). Similarly, in the emotional dimension, DA and 5-HT represent two poles of the horizontal dimension, which is the valence dimension, while the NE represents the vertical dimension, which means arousal (11). This model might be the first theory to connect monoamine neurotransmitters with basic emotions and emotional dimensions.

## Emotion-Based Interventions in Treating PSD

If patients are diagnosed with PSD, they are usually treated with antidepressants (183). However, the effectiveness of antidepressants in clinical practice is only approximately 50% (184). Therefore, it is necessary to provide additional effective and safe treatment for PSD. Emotional control for PSD showed great potential in the treatment and prevention of PSD in recent studies (7). The effects of several major psychotherapies in recent studies are summarized in the following section.

Cognitive reappraisal is an effective and common intervention therapy for the treatment of depression (185). In a recent meta-study, cognitive behavioral therapy (CBT) interventions yielded a larger short-term decrease in depression scores (126). CBT was also widely used in clinical treatment for PSD (186). A single-blind randomized controlled trial of PSD revealed that CBT was as effective as citalopram for late-onset post-ischemic depression and was more effective than rehabilitation alone (100). A recent meta-study also reported that both CBT alone and CBT with antidepressants all showed significantly improved depressive symptoms in PSD (18). However, most studies have not considered the time of depression onset. Hou et al. (187) found that antidepressants and psychological therapy may not improve the symptoms of depression in patients during the first 3 months. Gao et al. (100) also reported that the most positive results of CBT for treating post-ischemic stroke depression occurred 3 months later. It may be associated with the biological changes in the brain tissue caused by stroke. Therefore, CBT is effective in treating PSD, but this effect usually occurs after the biological changes in brain tissue stabilize. In addition, a recent study has demonstrated the relevance of the MAOA gene for the treatment outcome of CBT, while the MAOA gene plays a key role in the degradation of monoamines, especially 5-HT and NE (188).

In a recent study, mindfulness meditation showed potential benefits for PSD (189). Mindfulness is defined as a process of openly attending, with awareness, to one's present moment experience (190). Mindfulness meditation includes at least three components: improved attention control, enhanced emotion regulation, and altered self-awareness (191). In a recent randomized controlled trial, Wang et al. (189) revealed that mindfulness intervention had positive effects on depression, social wellbeing, and emotional wellbeing of patients with PSD.

In addition, other treatments of implicit emotional control were also recommended for patients with PSD, such as literature therapy and art treatment (192–194). Literature therapy is psychotherapeutic, which helps patients develop insight and awareness of negative thoughts and emotions, provides answers to problems, and supports them to practice these approaches in their daily life (195). Art treatment is defined as the therapeutic use of verbal treatment methods, using rhythms, sensory stimulation, symbolic motions, and colors that could facilitate the addressing of the patients' psychological issues (196). All of these treatments have been proved to work well for depression by changing the unconscious minds, which can be called implicit emotional control (194, 195, 197). Therefore, Eum et al. (195) suggested that literature therapy and art treatment could serve as a useful emotional control to help patients with stroke in their rehabilitation process (195).

Even though many studies have reported that emotional control has a great potential effect on PSD, there are still more questions that remain unanswered, e.g., the best time for emotional intervention in PSD. Most of the studies have not considered time, e.g., Hou et al. (187) found that antidepressants and psychological therapy only play a role after 3 months. In addition, there is no standard process for emotional control for PSD. Most researchers appealed to make a more individualized plan for different patients (193, 198). Therefore, a series of studies (18, 179) showed that the evidence for emotional control in PSD is still inconclusive.

## Conclusion And Perspectives

In this study, we briefly introduced the incidence, risk factors, and diagnosis of PSD. Then, we introduced the “monoamine hypothesis,” the role of three monoamines in PSD, and the antidepressant drugs primarily targeting these three monoamines and their receptors. Next, we elaborated on a new model of emotion based on the “monoamine hypothesis.” We hope to clarify the relationship between the three monoamines, emotion, and PSD. Patients with PSD have some changes in their microbiome and metabolism, and these potential biomarkers and microorganisms may aid in the diagnosis and treatment of the disease. Finally, since all drugs have side effects and the effectiveness of antidepressants in clinical practice is less than ~50%, we introduced some emotional controls for PSD. We hope this study could help with the diagnosis and treatment of PSD.

## Author Contributions

All authors listed have made a substantial, direct, and intellectual contribution to the work and approved it for publication.

## Funding

The study was supported by a grant from the Foundation of Humanities and Arts from the Ministry of Education in China (19YJAZH083) and by the National Nature Science Foundation in China (82101602 and 82171392).

## Conflict of Interest

The authors declare that the research was conducted in the absence of any commercial or financial relationships that could be construed as a potential conflict of interest.

## Publisher's Note

All claims expressed in this article are solely those of the authors and do not necessarily represent those of their affiliated organizations, or those of the publisher, the editors and the reviewers. Any product that may be evaluated in this article, or claim that may be made by its manufacturer, is not guaranteed or endorsed by the publisher.

## References

[1] Das J Rajanikanth GK. Post stroke depression: the sequelae of cerebral stroke. Neurosci Biobehav Rev. (2018) 90:104–14. 10.1016/j.neubiorev.2018.04.005 29656030

[2] Hackett ML Pickles K. Part I: frequency of depression after stroke: an updated systematic review and meta-analysis of observational studies. Int J Stroke. (2014) 9:1017–25. 10.1111/ijs.12357 25117911

[3] Chemerinski E Robinson RG Kosier JT. Improved Recovery in Activities of Daily Living Associated With Remission of Poststroke Depression. Stroke. (2001) 32:113–7. 10.1161/01.STR.32.1.113 11136924

[4] Loubinoux I Kronenberg G Endres M Schumann-Bard P Freret T Filipkowski RK. Post-stroke depression: mechanisms, translation and therapy. J Cell Mol Med. (2012) 16:1961–9. 10.1111/j.1582-4934.2012.01555.x 22348642 PMC3822966

[5] Williams LS Ghose SS Swindle RW. Depression other mental health diagnoses increase mortality risk after ischemic stroke. Am J Psychiatry. (2004) 161:1090–5. 10.1176/appi.ajp.161.6.1090 15169698

[6] Lin DJ Finklestein SP Cramer SC. New directions in treatments targeting stroke recovery. Stroke. (2018) 49:3107–14. 10.1161/STROKEAHA.118.021359 30571435 PMC6309806

[7] Paolucci S. Advances in antidepressants for treating post-stroke depression. Expert Opin Pharmacother. (2017) 18:1011–7. 10.1080/14656566.2017.1334765 28535081

[8] Krishnan V Nestler EJ. The molecular neurobiology of depression. Nature. (2008) 455:894–902. 10.1038/nature07455 18923511 PMC2721780

[9] Pittenger C Duman RS. Stress, depression, and neuroplasticity: a convergence of mechanisms. Neuropsychopharmacology. (2008) 33:88–109. 10.1038/sj.npp.1301574 17851537

[10] Berton O Nestler EJ. New approaches to antidepressant drug discovery: beyond monoamines. Nat Rev Neurosci. (2006) 7:137–51. 10.1038/nrn1846 16429123

[11] Liang F Xu Q Jiang M Feng R Jiang S Yuan B. Emotion Induced Monoamine Neuromodulator Release Affects Functional Neurological Disorders. Front Cell Dev Biol. (2021) 9:633048. 10.3389/fcell.2021.633048 33659255 PMC7917220

[12] Gu S Wang F Patel NP Bourgeois JA Huang JH. A Model for basic emotions using observations of behavior in drosophila. Front Psychol. (2019) 10:781. 10.3389/fpsyg.2019.00781 31068849 PMC6491740

[13] Gu S Wang W Wang F Huang JH. Neuromodulator emotion biomarker for stress induced mental disorders. Neural Plast. (2016) 2016:2609128. 10.1155/2016/2609128 27051536 PMC4808661

[14] Xu Q Jiang M Gu S Wang F Yuan B. Early life stress induced DNA methylation of monoamine oxidases leads to depressive-like behavior. Front Cell Dev Biol. (2020) 8:582247. 10.3389/fcell.2020.582247 33015076 PMC7505948

[15] Medeiros GC Roy D Kontos N Beach SR. Post-stroke depression: a 2020 updated review. Gen Hosp Psychiatry. (2020) 66:70–80. 10.1016/j.genhosppsych.2020.06.011 32717644

[16] Villa RF Ferrari F Moretti A. Post-stroke depression: Mechanisms and pharmacological treatment. Pharmacol Ther. (2018) 184:131–44. 10.1016/j.pharmthera.2017.11.005 29128343

[17] Shi Y Yang D Zeng Y Wu W. Risk factors for post-stroke depression: a meta-analysis. Front Aging Neurosci. (2017) 9:218. 10.3389/fnagi.2017.00218 28744213 PMC5504146

[18] Wang SB Wang YY Zhang QE Wu SL Ng CH Ungvari GS. Cognitive behavioral therapy for post-stroke depression: a meta-analysis. J Affect Disord. (2018) 235:589–96. 10.1016/j.jad.2018.04.011 29704854

[19] Zhang E Liao P. Brain-derived neurotrophic factor and post-stroke depression. J Neurosci Res. (2020) 98:537–48. 10.1002/jnr.24510 31385340

[20] Cumming TB Churilov L Skoog I Blomstrand C Linden T. Little evidence for different phenomenology in poststroke depression. Acta Psychiatr Scand. (2010) 121:424–30. 10.1111/j.1600-0447.2010.01558.x 20384602

[21] de Man-van Ginkel JM Hafsteinsdottir TB Lindeman E Geerlings MI Grobbee DE Schuurmans MJ. Clinical manifestation of depression after stroke: is it different from depression in other patient populations? PLoS ONE. (2015) 10:e0144450. 10.1371/journal.pone.0144450 26637178 PMC4670173

[22] Saunders BT Richard JM Margolis EB Janak PH. Dopamine neurons create Pavlovian conditioned stimuli with circuit-defined motivational properties. Nat Neurosci. (2018) 21:1072–83. 10.1038/s41593-018-0191-4 30038277 PMC6082399

[23] Robinson RG Jorge RE. Post-stroke depression: a review. Am J Psychiatry. (2016) 173:221–31. 10.1176/appi.ajp.2015.15030363 26684921

[24] Ayerbe L Ayis S Wolfe CD Rudd AG. Natural history, predictors and outcomes of depression after stroke: systematic review and meta-analysis. Br J Psychiatry. (2013) 202:14–21. 10.1192/bjp.bp.111.107664 23284148

[25] Bartoli F Di Brita C Crocamo C Clerici M Carra G. Early post-stroke depression and mortality: meta-analysis and meta-regression. Front Psychiatry. (2018) 9:530. 10.3389/fpsyt.2018.00530 30443225 PMC6221899

[26] Hackett ML Anderson CS. Predictors of depression after stroke: a systematic review of observational studies. Stroke. (2005) 36:2296–301. 10.1161/01.STR.0000183622.75135.a4 16179565

[27] Chen N-T Lin P-H Guo Y-LL. Long-term exposure to high temperature associated with the incidence of major depressive disorder. Science of The Total Environment. (2019) 659:1016–20. 10.1016/j.scitotenv.2018.12.434 31096317

[28] Werheid KA. Two-phase pathogenetic model of depression after stroke. Gerontology. (2015) 62:33–9. 10.1159/000381876 26113201

[29] Khedr EM Abdelrahman AA Desoky T Zaki AF Gamea A. Post-stroke depression: frequency, risk factors, and impact on quality of life among 103 stroke patients—hospital-based study. The Egyptian Journal of Neurology, Psychiatry and Neurosurgery. (2020) 56. 10.1186/s41983-020-00199-8

[30] Guo J Wang J Sun W Liu X. The advances of post-stroke depression: 2021 update. J Neurol. (2021). 10.1007/s00415-021-10597-4 34052887

[31] Douven E Kohler S Rodriguez MMF Staals J Verhey FRJ Aalten P. Imaging markers of post-stroke depression and apathy: a systematic review and meta-analysis. Neuropsychol Rev. (2017) 27:202–19. 10.1007/s11065-017-9356-2 28831649 PMC5613051

[32] Tecuta L Tomba E Grandi S Fava GA. Demoralization: a systematic review on its clinical characterization. Psychol Med. (2015) 45:673–91. 10.1017/S0033291714001597 25032712

[33] Petrova E Ponevejsky E Savina M Koltsova E. Post-stroke apathy. Consilium Medicum. (2020) 22:33–7. 10.26442/20751753.2020.9.200274

[34] Douven E Staals J Freeze WM Schievink SH Hellebrekers DM Wolz R. Imaging markers associated with the development of post-stroke depression and apathy: results of the cognition and affect after stroke - a prospective evaluation of risks study. Eur Stroke J. (2020) 5:78–84. 10.1177/2396987319883445 32232173 PMC7092734

[35] Manning KJ Taylor WD. Poststroke depression and apathy: why should we care? Am J Geriatr Psychiatry. (2020) 28:1210–2. 10.1016/j.jagp.2020.03.005 32303404

[36] McBurney-Lin J Lu J Zuo Y Yang H. Locus coeruleus-norepinephrine modulation of sensory processing and perception: a focused review. Neurosci Biobehav Rev. (2019) 105:190–9. 10.1016/j.neubiorev.2019.06.009 31260703 PMC6742544

[37] van Dalen JW Moll van Charante EP Nederkoorn PJ van Gool WA Richard E. Poststroke apathy. Stroke. (2013) 44:851–60. 10.1161/STROKEAHA.112.674614 23362076

[38] Selvaraj S Arora T Casameni Montiel T Grey I Alfraih H Fadipe M. Early screening for post-stroke depression, and the effect on functional outcomes, quality of life and mortality: a protocol for a systematic review and meta-analysis. BMJ Open. (2021) 11:e050451. 10.1136/bmjopen-2021-050451 34404715 PMC8372879

[39] Goodarzi ZS Mele BS Roberts DJ Holroyd-Leduc J. Depression Case Finding in Individuals with Dementia: A Systematic Review and Meta-Analysis. J Am Geriatr Soc. (2017) 65:937–48. 10.1111/jgs.14713 28152174

[40] Meader N Moe-Byrne T Llewellyn A Mitchell AJ. Screening for poststroke major depression: a meta-analysis of diagnostic validity studies. J Neurol Neurosurg Psychiatry. (2014) 85:198–206. 10.1136/jnnp-2012-304194 23385849

[41] Dajpratham P Pukrittayakamee P Atsariyasing W Wannarit K Boonhong J Pongpirul K. The validity and reliability of the PHQ-9 in screening for post-stroke depression. BMC Psychiatry. (2020) 20:291. 10.1186/s12888-020-02699-6 32517743 PMC7285729

[42] Jorgensen TS Wium-Andersen IK Wium-Andersen MK Jorgensen MB Prescott E Maartensson S. Incidence of depression after stroke, and associated risk factors and mortality outcomes, in a large cohort of Danish patients. JAMA Psychiatry. (2016) 73:1032–40. 10.1001/jamapsychiatry.2016.1932 27603000

[43] Vataja R Pohjasvaara T Leppävuori A Mäntylä R Aronen HJ Salonen O. (2001). Magnetic Resonance Imaging Correlates of Depression After Ischemic Stroke. Archives of General Psychiatry 58, 925–931. 10.1001/archpsyc.58.10.925 11576030

[44] De Ryck A Brouns R Geurden M Elseviers M De Deyn PP Engelborghs S. Risk factors for poststroke depression: identification of inconsistencies based on a systematic review. J Geriatr Psychiatry Neurol. (2014) 27:147–58. 10.1177/0891988714527514 24713406

[45] Ayerbe L Ayis S Rudd AG Heuschmann PU Wolfe CD. Natural history, predictors, and associations of depression 5 years after stroke: the South London Stroke Register. Stroke. (2011) 42:1907–11. 10.1161/STROKEAHA.110.605808 21566241

[46] Tsai CS Wu CL Hung TH Chou SY Su JA. Incidence risk factors of poststroke depression in patients with acute ischemic stroke: A 1-year prospective study in Taiwan. Biomed J. (2016) 39:195–200. 10.1016/j.bj.2015.10.004 27621121 PMC6140301

[47] Robinson RG Jorge RE Moser DJ Acion L Solodkin A Small SL. Escitalopram and problem-solving therapy for prevention of poststroke depression: a randomized controlled trial. JAMA. (2008) 299:2391–400. 10.1001/jama.299.20.2391 18505948 PMC2743160

[48] Robinson RG Kubos KL Starr LB Rao K Price TR. Mood disorders in stroke patients: importance of location of lesion. Brain. (1984) 107:81–93. 10.1093/brain/107.1.81 6697163

[49] Starkstein SE Robinson RG Price TR. Comparison of cortical and subcortical lesions in the production of poststroke mood disorders. Brain. (1987) 110:1045–59. 10.1093/brain/110.4.1045 3651794

[50] Jorge R Moser D Acion L Robinson R. Treatment of vascular depression using repetitive transcranial magnetic stimulation. Arch Gen Psychiatry. (2008) 65:268–76. 10.1001/archgenpsychiatry.2007.45 18316673

[51] Brown C Hasson H Thyselius V Almborg AH. Post-stroke depression and functional independence: a conundrum. Acta Neurol Scand. (2012) 126:45–51. 10.1111/j.1600-0404.2011.01595.x 21992112

[52] Tang WK Chen YK Lu JY Chu WC Mok VC Ungvari GS. Cerebral microbleeds and depression in lacunar stroke. Stroke. (2011) 42:2443–6. 10.1161/STROKEAHA.111.614586 21757672

[53] Li J Zhao YD Zeng JW Chen XY Wang RD Cheng SY. Serum Brain-derived neurotrophic factor levels in post-stroke depression. J Affect Disord. (2014) 168:373–9. 10.1016/j.jad.2014.07.011 25106034

[54] Carota A Berney A Aybek S Iaria G Staub F Ghika-Schmid F. A prospective study of predictors of poststroke depression. Neurology. (2005) 64:428–33. 10.1212/01.wnl.0000150935.05940.2d 15699370

[55] Ried LD Jia H Cameon R Feng H Wang X Tueth M. Does prestroke depression impact poststroke depression and treatment? Am J Geriatr Psychiatry. (2010) 18:624–33. 10.1097/JGP.0b013e3181ca822b 20220578

[56] Taylor-Rowan M Momoh O Ayerbe L Evans JJ Stott DJ Quinn TJ. Prevalence of pre-stroke depression and its association with post-stroke depression: a systematic review and meta-analysis. Psychol Med. (2019) 49:685–96. 10.1017/S0033291718002003 30107864

[57] Northcott S Moss B Harrison K Hilari K. A. systematic review of the impact of stroke on social support and social networks: associated factors and patterns of change. Clin Rehabil. (2016) 30:811–31. 10.1177/0269215515602136 26330297

[58] Liu Y Zhang D Luo Y. How disgust facilitates avoidance: an ERP study on attention modulation by threats. Soc Cogn Affect Neurosci. (2015) 10:598–604. 10.1093/scan/nsu094 24974395 PMC4381247

[59] Johnson J Minarik P Nyström K Bautista C Gorman M. Poststroke depression incidence and risk factors. J Neurosci Nurs. (2006) 38:316–27. 10.1097/01376517-200609000-00008 16989301

[60] Trofimova I. Functionality versus dimensionality in psychological taxonomies, and a puzzle of emotional valence. Philos Trans R Soc B Biol Sci. (2018) 373:20170167. 10.1098/rstb.2017.0167 29483351 PMC5832691

[61] Gu S Gao M Yan Y Wang F Tang Y Huang JH. The Neural Mechanism Underlying Cognitive and Emotional Processes in Creativity. Front Psychol. (2018) 9. 10.3389/fpsyg.2018.01924 PMC6220028 30429805

[62] Trofimova IN Gaykalova AA. Emotionality vs. other biobehavioural traits: A look at neurochemical biomarkers for their differentiation. Front Psychol. (2021) 12:781631. 10.3389/fpsyg.2021.781631 34987450 PMC8720768

[63] Santos M Kovari E Gold G Bozikas VP Hof PR Bouras C. The neuroanatomical model of post-stroke depression: towards a change of focus? J Neurol Sci. (2009) 283:158–62. 10.1016/j.jns.2009.02.334 19264329 PMC2915758

[64] Xekardaki A Santos M Hof P Kovari E Bouras C Giannakopoulos P. Neuropathological substrates and structural changes in late-life depression: the impact of vascular burden. Acta Neuropathol. (2012) 124:453–64. 10.1007/s00401-012-1021-5 22836715

[65] Dean J Keshavan M. The neurobiology of depression: an integrated view. Asian J Psychiatr. (2017) 27:101–11. 10.1016/j.ajp.2017.01.025 28558878

[66] Yagishita S. Transient sustained effects of dopamine and serotonin signaling in motivation-related behavior. Psychiatry Clin Neurosci. (2020) 74:91–8. 10.1111/pcn.12942 31599012

[67] Chong TTJ. Updating the role of dopamine in human motivation and apathy. Curr Opin Behav Sci. (2018) 22:35–41. 10.1016/j.cobeha.2017.12.010

[68] Correia PA Lottem E Banerjee D Machado AS Carey MR Mainen ZF. Transient inhibition and long-term facilitation of locomotion by phasic optogenetic activation of serotonin neurons. Elife. (2017) 6. 10.7554/eLife.20975 PMC5308893 28193320

[69] Howe MW Dombeck DA. Rapid signalling in distinct dopaminergic axons during locomotion and reward. Nature. (2016) 535:505–10. 10.1038/nature18942 27398617 PMC4970879

[70] Li Y Zhong W Wang D Feng Q Liu Z Zhou J. Serotonin neurons in the dorsal raphe nucleus encode reward signals. Nat Commun. (2016) 7:10503. 10.1038/ncomms10503 26818705 PMC4738365

[71] Yoshida K Drew MR Mimura M Tanaka KF. Serotonin-mediated inhibition of ventral hippocampus is required for sustained goal-directed behavior. Nat Neurosci. (2019) 22:770–7. 10.1038/s41593-019-0376-5 30988523

[72] Kawashima T. The role of the serotonergic system in motor control. Neurosci Res. (2018) 129:32–9. 10.1016/j.neures.2017.07.005 28774814

[73] de Jong JW Afjei SA Pollak Dorocic I Peck JR Liu C Kim CK. A neural circuit mechanism for encoding aversive stimuli in the mesolimbic dopamine system. Neuron. (2019) 101, 133–151 e137. 10.1016/j.neuron.2018.11.005 30503173 PMC6317997

[74] Troubat R Barone P Leman S Desmidt T Cressant A Atanasova B. Neuroinflammation and depression: a review. Eur J Neurosci. (2021) 53:151–71. 10.1111/ejn.14720 32150310

[75] Hillhouse TM Porter JH. A. brief history of the development of antidepressant drugs: from monoamines to glutamate. Exp Clin Psychopharmacol. (2015) 23:1–21. 10.1037/a0038550 25643025 PMC4428540

[76] Carvalho AF Sharma MS Brunoni AR Vieta E Fava GA. The safety, tolerability and risks associated with the use of newer generation antidepressant drugs: a critical review of the literature. Psychother Psychosom. (2016) 85:270–88. 10.1159/000447034 27508501

[77] Martins J Brijesh S. Phytochemistry pharmacology of anti-depressant medicinal plants: a review. Biomed Pharmacother. (2018) 104:343–65. 10.1016/j.biopha.2018.05.044 29778018

[78] Kraus C Castren E Kasper S Lanzenberger R. Serotonin neuroplasticity - Links between molecular, functional and structural pathophysiology in depression. Neurosci Biobehav Rev. (2017) 77:317–26. 10.1016/j.neubiorev.2017.03.007 28342763

[79] He Z Jiang Y Gu S Wu D Qin D Feng G. The aversion function of the limbic dopaminergic neurons and their roles in functional neurological disorders. Front Cell Dev Biol. (2021) 9:713762. 10.3389/fcell.2021.713762 34616730 PMC8488171

[80] Solati J Salari AA Bakhtiari A. 5HT(1A) and 5HT(1B) receptors of medial prefrontal cortex modulate anxiogenic-like behaviors in rats. Neurosci Lett. (2011) 504:325–9. 10.1016/j.neulet.2011.09.058 21982809

[81] Akizawa F Mizuhiki T Setogawa T Takafuji M Shidara M. The effect of 5-HT1A receptor antagonist on reward-based decision-making. J Physiol Sci. (2019) 69:1057–69. 10.1007/s12576-019-00725-1 31705485 PMC10717930

[82] Lu J Zhang C Lv J Zhu X Jiang X Lu W. Antiallergic drug desloratadine as a selective antagonist of 5HT2A receptor ameliorates pathology of Alzheimer's disease model mice by improving microglial dysfunction. Aging Cell. (2021) 20:e13286. 10.1111/acel.13286 33369003 PMC7811850

[83] Morrison TR Ricci LA Puckett AS Joyce J Curran R Davis C. Serotonin type-3 receptors differentially modulate anxiety and aggression during withdrawal from adolescent anabolic steroid exposure. Horm Behav. (2020) 119:104650. 10.1016/j.yhbeh.2019.104650 31805280

[84] Bockaert J Claeysen S Compan V Dumuis A. 5-HT4 receptors: History, molecular pharmacology and brain functions. Neuropharmacology. (2008) 55:922–31. 10.1016/j.neuropharm.2008.05.013 18603269

[85] Stockmeier CA. Involvement of serotonin in depression: evidence from postmortem and imaging studies of serotonin receptors and the serotonin transporter. J Psychiatr Res. (2003) 37:357–73. 10.1016/s0022-3956(03)00050-5 12849929

[86] Gao HQ Zhu HY Zhang YQ Wang LX. Reduction of cerebrospinal fluid and plasma serotonin in patients with post-stroke depression: a preliminary report. Clin Invest Med. (2008) 31:E351–356. 10.25011/cim.v31i6.4921 19032905

[87] Raison CL Borisov AS Majer M Drake DF Pagnoni G Woolwine BJ. Activation of central nervous system inflammatory pathways by interferon-alpha: relationship to monoamines and depression. Biol Psychiatry. (2009) 65:296–303. 10.1016/j.biopsych.2008.08.010 18801471 PMC2655138

[88] Xu Q Jiang M Gu S Zhang X Feng G Ma X. Metabolomics changes in brain-gut axis after unpredictable chronic mild stress. Psychopharmacology. (2022). 10.1007/s00213-021-05958-w PMC8891102 35133451

[89] Popova NK Ilchibaeva TV Naumenko VS. Neurotrophic Factors (BDNF and GDNF) and the Serotonergic System of the Brain. Biochemistry (Mosc). (2017) 82:308–17. 10.1134/S0006297917030099 28320272

[90] Naumenko VS Kondaurova EM Bazovkina DV Tsybko AS Tikhonova MA Kulikov AV. Effect of brain-derived neurotrophic factor on behavior and key members of the brain serotonin system in genetically predisposed to behavioral disorders mouse strains. Neuroscience. (2012) 214:59–67. 10.1016/j.neuroscience.2012.04.031 22531372

[91] Leonard BE. Inflammation depression: a causal or coincidental link to the pathophysiology? Acta Neuropsychiatr. (2018) 30:1–16. 10.1017/neu.2016.69 28112061

[92] Juruena MF Bocharova M Agustini B Young AH. Atypical depression and non-atypical depression: Is HPA axis function a biomarker? A systematic review. J Affect Disord. (2018) 233:45–67. 10.1016/j.jad.2017.09.052 29150144

[93] Fiksdal A Hanlin L Kuras Y Gianferante D Chen X Thoma MV. Associations between symptoms of depression and anxiety and cortisol responses to and recovery from acute stress. Psychoneuroendocrinology. (2019) 102:44–52. 10.1016/j.psyneuen.2018.11.035 30513499 PMC6420396

[94] Mayer SE Lopez-Duran NL Sen S Abelson JL. Chronic stress, hair cortisol and depression: A prospective and longitudinal study of medical internship. Psychoneuroendocrinology. (2018) 92:57–65. 10.1016/j.psyneuen.2018.03.020 29627713 PMC5924646

[95] Lang UE Hellweg R Gallinat J. BDNF serum concentrations in healthy volunteers are associated with depression-related personality traits. Neuropsychopharmacology. (2004) 29:795–8. 10.1038/sj.npp.1300382 14735133

[96] Li J Lu C Gao Z Feng Y Luo H Lu T. SNRIs achieve faster antidepressant effects than SSRIs by elevating the concentrations of dopamine in the forebrain. Neuropharmacology. (2020) 177:108237. 10.1016/j.neuropharm.2020.108237 32710978

[97] Xu XM Zou DZ Shen LY Liu Y Zhou XY Pu JC. Efficacy and feasibility of antidepressant treatment in patients with post-stroke depression. Medicine (Baltimore). (2016) 95:e5349. 10.1097/MD.0000000000005349 27828858 PMC5106064

[98] Boustani M Campbell N Munger S Maidment I Fox C. Impact of anticholinergics on the aging brain: a review and practical application. Aging health. (2008) 4:311–20. 10.2217/1745509x.4.3.311

[99] Choi-Kwon S Han SW Kwon SU Kang DW Choi JM Kim JS. Fluoxetine treatment in poststroke depression, emotional incontinence, and anger proneness: a double-blind, placebo-controlled study. Stroke. (2006) 37:156–61. 10.1161/01.STR.0000190892.93663.e2 16306470

[100] Gao J Lin M Zhao J Bi S Ni Z Shang X. Different interventions for post-ischaemic stroke depression in different time periods: a single-blind randomized controlled trial with stratification by time after stroke. Clin Rehabil. (2017) 31:71–81. 10.1177/0269215515626232 26817808

[101] Kim JS Lee E-J Chang D-I Park J-H Ahn SH Cha J-K. Efficacy of early administration of escitalopram on depressive and emotional symptoms and neurological dysfunction after stroke: a multicentre, double-blind, randomised, placebo-controlled study. Lancet Psychiat. (2017) 4:33–41. 10.1016/s2215-0366(16)30417-5 28012485

[102] Robinson RG Schultz SK Castillo C Kopel T Kosier JT Newman RM. Nortriptyline versus fluoxetine in the treatment of depression and in short-term recovery after stroke: a placebo-controlled, double-blind study. Am J Psychiatry. (2000) 157:351–9. 10.1176/appi.ajp.157.3.351 10698809

[103] Salter KL Foley NC Zhu L Jutai JW Teasell RW. Prevention of poststroke depression: does prophylactic pharmacotherapy work? J Stroke Cerebrovasc Dis. (2013) 22:1243–51. 10.1016/j.jstrokecerebrovasdis.2012.03.013 22554569

[104] Karaiskos D Tzavellas E Spengos K Vassilopoulou S Paparrigopoulos T. Duloxetine versus citalopram and sertraline in the treatment of poststroke depression, anxiety, and fatigue. J Neuropsychiatry Clin Neurosci. (2012) 24:349–53. 10.1176/appi.neuropsych.11110325 23037649

[105] Tan S Huang X Ding L Hong H. Efficacy safety of citalopram in treating post-stroke depression: a meta-analysis. Eur Neurol. (2015) 74:188–201. 10.1159/000441446 26559658

[106] Mortensen JK Johnsen SP Larsson H Andersen G. Early antidepressant treatment and all-cause 30-day mortality in patients with ischemic stroke. Cerebrovasc Dis. (2015) 40:81–90. 10.1159/000435819 26184925

[107] Mortensen JK Larsson H Johnsen SP Andersen G. Post stroke use of selective serotonin reuptake inhibitors and clinical outcome among patients with ischemic stroke: a nationwide propensity score-matched follow-up study. Stroke. (2013) 44:420–6. 10.1161/STROKEAHA.112.674242 23306326

[108] Tsai CS Wu CL Chou SY Tsang HY Hung TH Su JA. Prevention of poststroke depression with milnacipran in patients with acute ischemic stroke: a double-blind randomized placebo-controlled trial. Int Clin Psychopharmacol. (2011) 26:263–7. 10.1097/YIC.0b013e32834a5c64 21811172

[109] Zhang LS Hu XY Yao LY Geng Y Wei LL Zhang JH. Prophylactic effects of duloxetine on post-stroke depression symptoms: an open single-blind trial. Eur Neurol. (2013) 69:336–43. 10.1159/000345374 23549225

[110] Connolly KR Thase ME. Vortioxetine: a New Treatment for Major Depressive Disorder. Expert Opin Pharmacother. (2016) 17:421–31. 10.1517/14656566.2016.1133588 26679430

[111] Fallon BA Ahern DK Pavlicova M Slavov I Skritskya N Barsky AJ. A randomized controlled trial of medication and cognitive-behavioral therapy for hypochondriasis. Am J Psychiatry. (2017) 174:756–64. 10.1176/appi.ajp.2017.16020189 28659038 PMC5957509

[112] Knott V Mahoney C Kennedy S Evans K. EEG correlates of acute and chronic paroxetine treatment in depression. J Affect Disord. (2002) 69:241–9. 10.1016/S0165-0327(01)00308-1 12103473

[113] Wu KY Liu CY Hsiao MC. Six-month paroxetine treatment of premenstrual dysphoric disorder: continuous versus intermittent treatment protocols. Psychiatry Clin Neurosci. (2008) 62:109–14. 10.1111/j.1440-1819.2007.01785.x 18289149

[114] Altamura AC Caldiroli A Buoli M. Pharmacokinetic evaluation of fluvoxamine for the treatment of anxiety disorders. Expert Opin Drug Metab Toxicol. (2015) 11:649–60. 10.1517/17425255.2015.1021331 25728382

[115] Zheng W Xiang YQ Cai DB Yang XH Zhang L Zheng W. Adjunctive fluvoxamine for schizophrenia: a meta-analysis of randomized double-blind, placebo-controlled trials. J Clin Psychopharmacol. (2020) 40:386–90. 10.1097/JCP.0000000000001245 32618683

[116] De Vane CL Liston HL Markowitz JS. Clinical pharmacokinetics of sertraline. Clin Pharmacokinet. (2002) 41:1247–66. 10.2165/00003088-200241150-00002 12452737

[117] Wagner KD Robb AS Findling RL Jin J Gutierrez MM Heydorn WE. A Randomized placebo-controlled trial of citalopram for the treatment of major depression in children and adolescents. Am J Psychiatry. (2004) 161:1079–83. 10.1176/appi.ajp.161.6.1079 15169696

[118] Thomsen PH Ebbesen C Persson C. Long-term experience with citalopram in the treatment of adolescent OCD. J Am Acad Child Adolesc Psychiatry. (2001) 40:895–902. 10.1097/00004583-200108000-00010 11501688

[119] Pastoor D Gobburu J. Clinical pharmacology review of escitalopram for the treatment of depression. Expert Opinion on Drug Metabolism & Toxicology. (2014) 10:121–8. 10.1517/17425255.2014.863873 24289655

[120] Rapp W. Comparative trial of amitriptyline-N-oxide and amitriptyline in the treatment of out-patients with depressive syndromes. Acta Psychiatr Scand. (1978) 58:245–55. 10.1111/j.1600-0447.1978.tb06936.x 360779

[121] Nierenberg A Papakostas G Petersen T Kelly K Iacoviello B Worthington J. Nortriptyline for Treatment-Resistant Depression. J Clin Psychiatry. (2003) 64:35–9. 10.4088/JCP.v64n0108 12590621

[122] Koran LM Aboujaoude E Ward H Shapira NA Sallee FR Gamel N. Pulse-loaded intravenous clomipramine in treatment-resistant obsessive-compulsive disorder. J Clin Psychopharmacol. (2006) 26:79–83. 10.1097/01.jcp.0000195112.24769.b3 16415712

[123] Nakagawa A Watanabe N Omori IM Barbui C Cipriani A McGuire H. Efficacy and tolerability of milnacipran in the treatment of major depression in comparison with other antidepressants. CNS Drugs. (2008) 22:587–602. 10.2165/00023210-200822070-00004 18547127

[124] Torres-Sanchez S Perez-Caballero L Mico JA Elorza J Berrocoso E. Preclinical discovery of duloxetine for the treatment of depression. Expert Opin Drug Discov. (2012) 7:745–55. 10.1517/17460441.2012.693912 22680253

[125] Davis L Pilkinton P Lin C Parker P Estes S Bartolucci A. A randomized, placebo-controlled trial of mirtazapine for the treatment of posttraumatic stress disorder in veterans. J Clin Psychiatry. (2020) 81. 10.4088/JCP.20m13267 33084254

[126] Lopez-Lopez JA Davies SR Caldwell DM Churchill R Peters TJ Tallon D. The process and delivery of CBT for depression in adults: a systematic review and network meta-analysis. Psychol Med. (2019) 49:1937–47. 10.1017/S003329171900120X 31179960 PMC6712954

[127] Furukawa TA Cipriani A Cowen PJ Leucht S Egger M Salanti G. Optimal dose of selective serotonin reuptake inhibitors, venlafaxine, and mirtazapine in major depression: a systematic review and dose-response meta-analysis. The Lancet Psychiatry. (2019) 6:601–9. 10.1016/s2215-0366(19)30217-2 31178367 PMC6586944

[128] Yeung WF Chung KF Yung KP Ng TH. Doxepin for insomnia: a systematic review of randomized placebo-controlled trials. Sleep Med Rev. (2015) 19:75–83. 10.1016/j.smrv.2014.06.001 25047681

[129] Menegas W Bergan JF Ogawa SK Isogai Y Umadevi Venkataraju K Osten P. Dopamine neurons projecting to the posterior striatum form an anatomically distinct subclass. Elife. (2015) 4:e10032. 10.7554/eLife.10032 26322384 PMC4598831

[130] Morales M Margolis EB. Ventral tegmental area: cellular heterogeneity, connectivity and behaviour. Nat Rev Neurosci. (2017) 18:73–85. 10.1038/nrn.2016.165 28053327

[131] Lammel S Hetzel A Hackel O Jones I Liss B Roeper J. Unique properties of mesoprefrontal neurons within a dual mesocorticolimbic dopamine system. Neuron. (2008) 57:760–73. 10.1016/j.neuron.2008.01.022 18341995

[132] Xia QP Cheng ZY He L. The modulatory role of dopamine receptors in brain neuroinflammation. Int Immunopharmacol. (2019) 76:105908. 10.1016/j.intimp.2019.105908 31622861

[133] Bagot RC Parise EM Pena CJ Zhang HX Maze I Chaudhury D. Ventral hippocampal afferents to the nucleus accumbens regulate susceptibility to depression. Nat Commun. (2015) 6:7062. 10.1038/ncomms8062 25952660 PMC4430111

[134] Du Y Liang H Zhang L Fu F. Administration of Huperzine A exerts antidepressant-like activity in a rat model of post-stroke depression. Pharmacol Biochem Behav. (2017) 158:32–8. 10.1016/j.pbb.2017.06.002 28583576

[135] Seo JS Wei J Qin L Kim Y Yan Z Greengard Cellular P and molecular basis for stress-induced depression. Mol Psychiatry. (2017) 22:1440–7. 10.1038/mp.2016.118 27457815 PMC5269558

[136] Whitton AE Reinen JM Slifstein M Ang YS McGrath PJ Iosifescu DV. Baseline reward processing and ventrostriatal dopamine function are associated with pramipexole response in depression. Brain. (2020) 143:701–10. 10.1093/brain/awaa002 32040562 PMC7009463

[137] Guessoum SB Le Strat Y Dubertret C Mallet J. A transnosographic approach of negative symptoms pathophysiology in schizophrenia and depressive disorders. Prog Neuropsychopharmacol Biol Psychiatry. (2020) 99:109862. 10.1016/j.pnpbp.2020.109862 31927053

[138] Chaudhury D Walsh JJ Friedman AK Juarez B Ku SM Koo JW. Rapid regulation of depression-related behaviours by control of midbrain dopamine neurons. Nature. (2013) 493:532–6. 10.1038/nature11713 23235832 PMC3554860

[139] Grace AA. Dysregulation of the dopamine system in the pathophysiology of schizophrenia and depression. Nat Rev Neurosci. (2016) 17:524–32. 10.1038/nrn.2016.57 27256556 PMC5166560

[140] Loubinoux I Demain B Davoust C Plas B Vaysse L. Stem cells and motor recovery after stroke. Ann Phys Rehabil Med. (2014) 57:499–508. 10.1016/j.rehab.2014.08.008 25282583

[141] Stahl SM. dextromethorphan/bupropion: a novel oral nmda (n-methyl-d-aspartate) receptor antagonist with multimodal activity. CNS Spectr. (2019) 24:461–6. 10.1017/S1092852919001470 31566163

[142] Schwarz LA Luo L. Organization of the locus coeruleus-norepinephrine system. Curr Biol. (2015) 25:R1051–6. 10.1016/j.cub.2015.09.039 26528750

[143] Maletic V Eramo A Gwin K Offord SJ Duffy RA. The Role of Norepinephrine and Its alpha-Adrenergic Receptors in the Pathophysiology and Treatment of Major Depressive Disorder and Schizophrenia: A Systematic Review. Front Psychiatry. (2017) 8:42. 10.3389/fpsyt.2017.00042 28367128 PMC5355451

[144] Ramos BP Arnsten AF. Adrenergic pharmacology and cognition: focus on the prefrontal cortex. Pharmacol Ther. (2007) 113:523–36. 10.1016/j.pharmthera.2006.11.006 17303246 PMC2151919

[145] Nabavi SM Daglia M Braidy N Nabavi SF. Natural products, micronutrients, and nutraceuticals for the treatment of depression: a short review. Nutr Neurosci. (2017) 20:180–94. 10.1080/1028415X.2015.1103461 26613119

[146] Watanabe I Li GY Imamura Y Nabeta H Kunitake Y Ishii H. Association of saliva 3-methoxy-4-hydroxyphenylglycol levels and a later depressive state in older subjects living in a rural community: 3-year follow-up study. Int J Geriatr Psychiatry. (2012) 27:321–6. 10.1002/gps.2729 21538541

[147] Leonard BE. Stress, norepinephrine and depression. Acta Neuropsychiatr. (2002) 14:173–80. 10.1034/j.1601-5215.2002.140403.x 26984329

[148] Zieglgänsberger W. Substance P and pain chronicity. Cell Tissue Res. (2019) 375:227–41. 10.1007/s00441-018-2922-y 30284083 PMC6335504

[149] Katsouni E Sakkas P Zarros A Skandali N Liapi C. The involvement of substance P in the induction of aggressive behavior. Peptides. (2009) 30:1586–91. 10.1016/j.peptides.2009.05.001 19442694

[150] Kinsman B Simmonds S Browning K Wenner M Farquhar W Stocker S. Integration of hypernatremia and angiotensin II by the organum vasculosum of the lamina terminalis regulates thirst. The Journal of Neuroscience. (2020) 40:2208–19. 10.1523/JNEUROSCI.2208-19.2020 32005766 PMC7055145

[151] Cera N Vargas-Cáceres S Oliveira C Monteiro J Branco D Pignatelli D. How relevant is the systemic oxytocin concentration for human sexual behavior? A systematic review. Sexual Medicine. (2021) 9:100370. 10.1016/j.esxm.2021.100370 34118520 PMC8360917

[152] Thul TA Corwin EJ Carlson NS Brennan PA Young LOxytocin J and postpartum depression: A systematic review. Psychoneuroendocrinology. (2020) 120:104793–104793. 10.1016/j.psyneuen.2020.104793 32683141 PMC7526479

[153] Ramos A Tufik S Troncone L. Control of Stress-Induced ACTH Secretion by Vasopressin and CRH: Additional Evidence. Neuropsychobiology. (2016) 73:184–90. 10.1159/000445480 27221315

[154] Tokarz V MacDonald P Klip A. The cell biology of systemic insulin function. J Cell Biol. (2018) 217, jcb.201802095. 10.1083/jcb.201802095 PMC6028526 29622564

[155] Miani A. Sexual arousal and rhythmic synchronization: a possible effect of vasopressin. Med Hypotheses. (2016) 93:122–5. 10.1016/j.mehy.2016.05.030 27372870

[156] Christ-Crain M. Vasopressin copeptin in health and disease. Rev Endocr Metab Disord. (2019) 20:1–12. 10.1007/s11154-019-09509-9 31656992

[157] Dray A Perkins M Bradykinin inflamMatory pain. Trends Neurosci. (1993) 16:99–104. 10.1016/0166-2236(93)90133-7 7681240

[158] Cooper S Dourish C Clifton P. CCK antagonists and CCK-monoamine interactions in the control of satiety. Am J Clini Nutr. (1992) 55:291S−295S. 10.1093/ajcn/55.1.291s 1728842

[159] Moran T Schwartz G. Neurobiology of cholecystokinin. Crit Rev Neurobiol. (1994) 9:1–28. 8828002

[160] Larsen CM Grattan D. Prolactin, neurogenesis, maternal behaviors. Brain Behav Immun. (2011) 26:201–9. 10.1016/j.bbi.2011.07.233 21820505

[161] Zhang H Su Q Yao D Wang S Dang S Ding D. Prolactin,a potential mediator of reduced social interactive behavior in newborn infants following maternal perinatal depressive symptoms. J Affect Disord. (2017) 215. 10.1016/j.jad.2017.03.029 28359983

[162] Joseph-Bravo P Jaimes-Hoy L Charli j.-l. Advances in TRH signaling. Rev Endocr Metab Disord. (2016) 17. 10.1007/s11154-016-9375-y 27515033

[163] Giuliano F Droupy S. Sexual side effects of pharmacological treatments. Progrès en urologie: journal de l'Association française d'urologie et de la Société française d'urologie. (2013) 23:804–10. 10.1016/j.purol.2013.01.008 23830275

[164] Majumdar I Weber H. Biology pharmacology of bombesin receptor subtype-3. Curr Opin Endocrinol Diabetes Obes. (2011) 19:3–7. 10.1097/MED.0b013e32834ec77d 22157398

[165] Dominguez-Lopez S Piccart E Lynch W Wollet M Sharpe A Beckstead M. Antagonism of neurotensin receptors in the ventral tegmental area decreases methamphetamine self-administration and methamphetamine seeking in mice. Int J Neuropsychopharmacol. (2017) 21. 10.1093/ijnp/pyx117 PMC5888879 29272412

[166] Francois A Low S Sypek E Christensen A Sotoudeh C Beier K. A Brainstem-Spinal Cord Inhibitory Circuit for Mechanical Pain Modulation by GABA and Enkephalins. Neuron. (2017) 93. 10.1016/j.neuron.2017.01.008 PMC7354674 28162807

[167] Smith KS Berridge KC Aldridge JW. Disentangling pleasure from incentive salience and learning signals in brain reward circuitry. Proc. Natl. Acad. Sci. U.S.A. (2011) 108:10935. 21670308 10.1073/pnas.1101920108 PMC3131314

[168] Davis G. Endorphins pain. Psychiatric Clinics of North America. (1983) 6:473–87. 10.1016/S0193-953X(18)30819-0 6316302

[169] Hawkes C. Endorphins: the basis of pleasure? J Neurol Neurosurg Psychiatry. (1992) 55:247–50. 10.1136/jnnp.55.4.247 1316428 PMC489033

[170] Obal F Krueger J. Biochemical regulation of non-rapid-eye-movement sleep. Front Biosci. (2003) 8:d520–550. 10.2741/1033 12700031

[171] Carr K. Effects of antibodies to dynorphin A and β-endorphin on lateral hypothalamic self-stimulation in ad libitum fed and food-deprived rats. Brain Res. (1990) 534:8–14. 10.1016/0006-8993(90)90106-L 1981487

[172] MacKinnon D Zamoiski R. Panic comorbidity with bipolar disorder: what is the manic-panic connection? Bipolar Disord. (2007) 8:648–64. 10.1111/j.1399-5618.2006.00356.x 17156152

[173] Marcinkiewcz C Mazzone C D'Agostino G Halladay L Hardaway A DiBerto J. Serotonin engages an anxiety and fear-promoting circuit in the extended amygdala. Nature. (2016) 537. 10.1038/nature19318 PMC5124365 27556938

[174] Alhadeff A Su Z Hernandez E Klima M Phillips S Holland R. A neural circuit for the suppression of pain by a competing need state. Cell. (2018) 173:140–152.e115. 10.1016/j.cell.2018.02.057 29570993 PMC5877408

[175] Gu S Wang F Cao C Wu E Tang YY Huang JH. an integrative way for studying neural basis of basic emotions with fMRI. Front Neurosci. (2019) 13. 10.3389/fnins.2019.00628 PMC6593191 31275107

[176] Ekman P. Emotions inside out. 130 Years after Darwin's “the expression of the emotions in man and animal”. Ann N Y Acad Sci. (2003) 1000:1–6. 10.1196/annals.1280.002 15045707

[177] Jack RE Garrod OGB Schyns PG. Dynamic facial expressions of emotion transmit an evolving hierarchy of signals over time. Curr Biol. (2014) 24:187–92. 10.1016/j.cub.2013.11.064 24388852

[178] Wang F Pereira A. Neuromodulation emotional feelings and affective disorders. Mens Sana Monogr. (2016) 14:5–29. 10.4103/0973-1229.154533 28031622 PMC5179628

[179] Wang F Yang J Pan F Ho RC Huang JH. Editorial: neurotransmitters and emotions. Front Psychol. (2020) 11:21. 10.3389/fpsyg.2020.00021 32116891 PMC7025515

[180] Russell JA. Core affect and the psychological construction of emotion. Psychol Rev. (2003) 110:145–72. 10.1037/0033-295x.110.1.145 12529060

[181] Moons WG Eisenberger NI Taylor SE. Anger and fear responses to stress have different biological profiles. Brain Behav Immun. (2010) 24:215–9. 10.1016/j.bbi.2009.08.009 19732822

[182] LeDoux JE Brown R. A higher-order theory of emotional consciousness. Proc Natl Acad Sci U S A. (2017) 114:E2016–25. 10.1073/pnas.1619316114 28202735 PMC5347624

[183] Olukolade O Osinowo HO. Efficacy of cognitive rehabilitation therapy on poststroke depression among survivors of first stroke attack in Ibadan, Nigeria. Behav Neurol. (2017) 2017:4058124. 10.1155/2017/4058124 28720980 PMC5504925

[184] Trivedi MH Rush AJ Wisniewski SR Nierenberg AA Warden D Ritz L. Evaluation of outcomes with citalopram for depression using measurement-based care in STAR^*^D: implications for clinical practice. Am J Psychiatry. (2006) 163:28–40. 10.1176/appi.ajp.163.1.28 16390886

[185] Uchida H Hiragaki Y Nishi Y Nakahara S Koumoto J Onmyoji Y. An iPad application-based intervention for improving post-stroke depression symptoms in a convalescent rehabilitation ward: a pilot randomized controlled clinical trial protocol. Internet Interv. (2020) 21:100340. 10.1016/j.invent.2020.100340 32944505 PMC7481559

[186] Kootker JA Rasquin SM Lem FC van Heugten CM Fasotti L Geurts AC. Augmented Cognitive Behavioral Therapy for Poststroke Depressive Symptoms: A Randomized Controlled Trial. Arch Phys Med Rehabil. (2017) 98:687–94. 10.1016/j.apmr.2016.10.013 27847195

[187] Hou W-H Liang H-W Hsieh C-L Hou C-Y Wen P-C Li C-Y. Effects of Stroke Rehabilitation on Incidence of Poststroke Depression: A Population-Based Cohort Study. J Clin Psychiatry. (2013) 74:e859–66. 10.4088/JCP.12m08259 24107772

[188] Trautmann S Richter J Muehlhan M Höfler M Wittchen H-U Domschke K. Does prior traumatization affect the treatment outcome of CBT for panic disorder? The potential role of the MAOA gene and depression symptoms. Eur Arch Psychiatry Clin Neurosci. (2019) 269:161–70. 10.1007/s00406-017-0823-9 28712090

[189] Wang X Li J Wang C Lv J. The effects of mindfulness-based intervention on quality of life and poststroke depression in patients with spontaneous intracerebral hemorrhage in China. Int J Geriatr Psychiatry. (2020) 35:572–80. 10.1002/gps.5273 32011785

[190] Creswell JD. Mindfulness interventions. Annu Rev Psychol. (2017) 68:491–516. 10.1146/annurev-psych-042716-051139 27687118

[191] Tang YY Holzel BK Posner MI. The neuroscience of mindfulness meditation. Nat Rev Neurosci. (2015) 16:213–25. 10.1038/nrn3916 25783612

[192] Eum Y Yim Literature J and art therapy in post-stroke psychological disorders. Tohoku J Exp Med. (2015) 235:17–23. 10.1620/tjem.235.17 25744067

[193] Grau-Sanchez J Duarte E Ramos-Escobar N Sierpowska J Rueda N Redon S. (2018). Music-supported therapy in the rehabilitation of subacute stroke patients: a randomized controlled trial. Ann N Y Acad Sci. 10.1111/nyas.13590 29607506

[194] Kim DS Park YG Choi JH Im SH Jung KJ Cha YA. Effects of music therapy on mood in stroke patients. Yonsei Med J. (2011) 52:977–81. 10.3349/ymj.2011.52.6.977 22028163 PMC3220261

[195] Eum Y Yim J Choi W. Elderly health and literature therapy: a theoretical review. Tohoku J Exp Med. (2014) 232:79–83. 10.1620/tjem.232.79 24522118

[196] Priebe S Savill M Bentall R Lauber C Reininghaus U McCrone P. Clinical effectiveness and cost-effectiveness of body psychotherapy in the treatment of negative symptoms of schizophrenia: a multicentre randomised controlled trial. Health Technology Assessment (Winchester, England). (2016) 20. 10.3310/hta20110 PMC4782808 26869182

[197] Hsu WC Lai HL. Effects of music on major depression in psychiatric inpatients. Arch Psychiatr Nurs. (2004) 18:193–9. 10.1016/j.apnu 15529285

[198] Broomfield NM Laidlaw K Hickabottom E Murray MF Pendrey R Whittick JE. Post-stroke depression: the case for augmented, individually tailored cognitive behavioural therapy. Clin Psychol Psychother. (2011) 18:202–17. 10.1002/cpp.711 20632301

